# An Ontology to Improve Transparency in Case Definition and Increase Case Finding of Infectious Intestinal Disease: Database Study in English General Practice

**DOI:** 10.2196/medinform.7641

**Published:** 2017-09-28

**Authors:** Simon de Lusignan, Stacy Shinneman, Ivelina Yonova, Jeremy van Vlymen, Alex J Elliot, Frederick Bolton, Gillian E Smith, Sarah O'Brien

**Affiliations:** ^1^ Section of Clinical Medicine and Ageing Department of Clinical and Experimental Medicine University of Surrey Guildford United Kingdom; ^2^ Royal College of General Practitioners Research and Surveillance Centre London United Kingdom; ^3^ Real-time Syndromic Surveillance Team National Infection Service Public Health England Birmingham United Kingdom; ^4^ Epidemiology and Population Health University of Liverpool Liverpool United Kingdom; ^5^ Institute of Psychology Health and Society University of Liverpool Liverpool United Kingdom

**Keywords:** dysentery, enteritis, enterobacteriaceae, enterocolitis, gastritis, gastroenteritis, intestinal diseases, medical records systems, computerized, norovirus, primary health care

## Abstract

**Background:**

Infectious intestinal disease (IID) has considerable health impact; there are 2 billion cases worldwide resulting in 1 million deaths and 78.7 million disability-adjusted life years lost. Reported IID incidence rates vary and this is partly because terms such as “diarrheal disease” and “acute infectious gastroenteritis” are used interchangeably. Ontologies provide a method of transparently comparing case definitions and disease incidence rates.

**Objective:**

This study sought to show how differences in case definition in part account for variation in incidence estimates for IID and how an ontological approach provides greater transparency to IID case finding.

**Methods:**

We compared three IID case definitions: (1) Royal College of General Practitioners Research and Surveillance Centre (RCGP RSC) definition based on mapping to the Ninth International Classification of Disease (ICD-9), (2) newer ICD-10 definition, and (3) ontological case definition. We calculated incidence rates and examined the contribution of four supporting concepts related to IID: symptoms, investigations, process of care (eg, notification to public health authorities), and therapies. We created a formal ontology using ontology Web language.

**Results:**

The ontological approach identified 5712 more cases of IID than the ICD-10 definition and 4482 more than the RCGP RSC definition from an initial cohort of 1,120,490. Weekly incidence using the ontological definition was 17.93/100,000 (95% CI 15.63-20.41), whereas for the ICD-10 definition the rate was 8.13/100,000 (95% CI 6.70-9.87), and for the RSC definition the rate was 10.24/100,000 (95% CI 8.55-12.12). Codes from the four supporting concepts were generally consistent across our three IID case definitions: 37.38% (3905/10,448) (95% CI 36.16-38.5) for the ontological definition, 38.33% (2287/5966) (95% CI 36.79-39.93) for the RSC definition, and 40.82% (1933/4736) (95% CI 39.03-42.66) for the ICD-10 definition. The proportion of laboratory results associated with a positive test result was 19.68% (546/2775).

**Conclusions:**

The standard RCGP RSC definition of IID, and its mapping to ICD-10, underestimates disease incidence. The ontological approach identified a larger proportion of new IID cases; the ontology divides contributory elements and enables transparency and comparison of rates. Results illustrate how improved diagnostic coding of IID combined with an ontological approach to case definition would provide a clearer picture of IID in the community, better inform GPs and public health services about circulating disease, and empower them to respond. We need to improve the Pathology Bounded Code List (PBCL) currently used by laboratories to electronically report results. Given advances in stool microbiology testing with a move to nonculture, PCR-based methods, the way microbiology results are reported and coded via PBCL needs to be reviewed and modernized.

## Introduction

### Background

The burden of infectious intestinal disease (IID) is considerable. The World Health Organization (WHO) estimated that foodborne disease from 22 pathogens accounted for 22 diseases resulted in 2 billion cases, over 1 million deaths, and 78.7 million disability-adjusted life years in 2010 [1]. The IID in the United Kingdom (IID2 study) [2] reported 274 cases per 1000 person-years, with 17.7 (95% CI 14.4-21.8) presenting to primary care. However, this may be an underestimate. Less restrictive – more representative (of coding practice) diagnostic criteria would greatly increase, for example, their estimate of norovirus by 26% to 59/1000 (95% CI 52.32-64.98) person-years equating to 3.7 (3.3-4.1) million infections annually [3].

Reported incidence rates for IID vary between 0.5% and 20% annually in the developed world [4-9]. Variation can be greatly attributed to underreporting and data types used to calculate rates [10]. Data used to report IID rates include: primary care records, hospital and other secondary care settings, prospective and retrospective surveys or questionnaires, notifications of disease to authorities, and reports of laboratory detection of pathogens [7,11,12]. Studies have concluded that approximately 1 in 20 IID patients in the community consult a general practitioner (GP) [7,13,14], hence incidence rates calculated based on primary care data are 0.5-3.3%—much lower than rates calculated with other methods [4,13,14].

Published variations may also be caused by imprecise or interchangeable use of the terms such as “diarrheal disease,” “acute infectious gastroenteritis” and “IID” and differing methods for describing cases, underscoring the importance of transparency when defining the disease [6,15]. The more general term “diarrheal disease” is used by the WHO and others in international public health as a symptom-based definition: infectious diarrhea and/or vomiting [6,11,16,17]. The terms “IID” and “acute gastroenteritis” tend to be more limited terms used to define patients with loose stools and/or vomiting for specific time periods and excluding chronic infections. Generally IID is defined as lasting less than 2 weeks, in the absence of known noninfectious causes, preceded by 2-3 symptom-free weeks [14,15]. Many studies list pathogens in their definition of IID or acute gastroenteritis; chronic or systemic conditions such as typhoid/paratyphoid and *Helicobacter* infections are often excluded [2,13].

Ontologies provide a method of systematically and transparently defining concepts and their relationships. They are used to clarify case finding and more accurately calculate disease incidence based on disease definitions that balance sensitivity and specificity [18,19]. In this study, we used a three-layer approach developed previously by the University of Surrey to develop an IID ontology [18]; we then used the ontological definition to calculate the incidence rate. The three-layer approach, an iterative process, includes development of disease concepts into an ontology, code collection, and logical data extraction [20].

UK general practice is highly computerized. Electronic registration–based systems ensure accurate denominators, and data from general practice provide opportunities for health research [21,22]. Most consultations are recorded on computers with key data—diagnosis, symptoms, investigative tests, and treatments—using a system called the Read codes [23]. The majority of UK practices are electronically connected to pathology laboratories, with generalized pathology results coded back into clinical records. Any laboratory results indicating pathogen detection should be coded directly by the clinician.

### Objectives

We aimed to test new technologies that provide general practitioners near real-time test results for a wide range of pathogens associated with IID [24]. We carried out this analysis to determine IID incidence from routine data using an ontological approach to make case finding more transparent and allow comparisons to other studies and data. We compared rates calculated using standard Royal College of General Practitioners (RCGP) Research and Surveillance Centre (RSC) and ICD-10 definitions with an ontological approach and reported impact on incidence rate.

## Methods

### IID Case Definition

We reviewed common IID case definitions published in the literature and standard coding systems used to record IID diagnoses in primary care settings and chose three IID definitions (Textbox 1).

**Description of IID case definitions chosen for this study.**

RSC definition

- Based on WHO’s International Classification of Diseases, ICD-8/9 versions, infectious intestinal diseases chapter
- Used for RCGP RSC weekly returns report
- Includes all codes falling into the infectious intestinal disease group of infectious and parasitic diseases within the concept hierarchies of the 5-byte Version 2 read Code system (A00-A09 codes)

ICD-10 definition

- Based on WHO’s International Classification of Diseases, ICD-10 version, infectious intestinal diseases chapter, all of which fall within A00-A09 chapter. More limited than the RSC definition
- Subset of ICD-8/9 and RSC definition due to exclusion of codes such as Helicobacter, nonintestinal Salmonella infections, Astrovirus, Calicivirus, and redundant codes

Ontological definition

- Based on IID case definition used during the Second Study of Infectious Intestinal Disease in the Community (IID2 Study)
- Includes all codes within the restricted ICD-10 definition, plus additional diagnostic codes that directly or partially map to IID2 case definition even though they fall outside of A00-A09 infectious intestinal disease group. Investigation and process of care codes that directly map to the case definition are also included
- The codes do not all necessarily fall into the A00-A09 infectious intestinal disease hierarchy used by the RSC and ICD-10 to define IID. This definition was based on the established case definition and was developed using an ontological approach designed to include all definite and possible IID cases recorded by clinicians in the RSC network

In the United Kingdom, the RCGP RSC case definition used for calculating weekly incidence of IID for the RSC’s weekly communicable and respiratory disease report is the established “gold standard” for surveillance [14,25]. IID incidence rates for the RSC weekly report are generated using codes from the IID chapter of Read codes version 2 (5-byte set), the GP coding system most commonly used in primary care since 1985 to enter data into electronic health records. The RSC definition includes Read codes for conditions in WHO’s International Classification of Diseases ICD-8/9 infectious intestinal diseases chapter (A00-A09 codes) [25]. To maintain consistency while monitoring long-term year-over-year trends in infections and outbreaks, RSC has conducted IID surveillance following the ICD-9 infectious intestinal diseases chapter, and as a result, many conditions not currently included in the newer WHO ICD-10 definition of IID continued to be included in the weekly returns report after ICD-10 was released [25]. To examine coding differences and relationships, we mapped IID codes between three ICD classifications and back to RSC weekly report codes.

For the ontological case definition of IID, we selected the more restrictive, well-documented case definition used during the Second Study of Infectious Intestinal Disease in the Community (IID2 Study), an extensively published, longitudinal study of IID incidence carried out in UK primary care [2,14,26]. The study defines IID as an infectious intestinal condition always causing diarrhea and sometimes other symptoms such as vomiting or nausea lasting 2 weeks or less [26].

### IID Ontology Development and Code Mapping

We used a three-level approach previously developed by the University of Surrey to establish an ontology based on IID case definition [20]. We formalized this ontology using Protégé, which is supported by grant GM10331601 from the National Institute of General Medical Sciences of the US National Institutes of Health [27].

The design of the ontology followed the structure used in problem-orientated records (POMR) and their associated coding system. This has its roots in the work of Lawrence Weed who created the idea of separating subjective (history) from objective (findings) and analysis (often diagnosis or problem) from plan (prescription or treatment). This is known internationally as Weed’s SOAP [28-30]. The classes in our ontology (Multimedia Appendix 1) broadly followed the components of SOAP: subjective (S), clinical features; objective (O), findings from laboratory tests, but could include objective clinical features such as fever if measured; analysis (A), the problem title or diagnoses; and plan (P), which includes the process of care code (which are often nonspecific) and an prescription or referral for further care. The computerized medical record (CMR) systems in the United Kingdom were historically strictly problem orientated, though those that are now in ascendency are more episode orientated [31]. The coding systems used within these systems have historically been hierarchical and used “chapters” that fit with the POMR structure [23].

We applied the ontology to the Read Code list by searching for codes indicative of IID diagnosis and mapped each into one of the following three classes [18,32]. Complete ontology and code lists are presented in supplementary tables (Multimedia Appendices 1 and 2).

1. **Direct mapping class:** All codes included in the direct mapping class indicate a clinician’s intention to record a definite IID diagnosis. Diagnostic codes fall into WHO’s ICD-10 infectious intestinal diseases chapter (A00-A09 codes) [25] and the infectious intestinal disease group of infectious and parasitic diseases within concept hierarchies of the 5-byte version 2 Read code system. Additional codes relate to investigative tests indicating laboratory detection of IID pathogens and processes of care indicating notification of IID.
2. **Partial mapping class:** All codes classified as partially mapping indicate a probable case of IID. These codes fall into the infectious intestinal disease group or other groups including gastrointestinal symptoms and other bacterial/infectious/ parasitic/digestive diseases. Additional codes relate to general IID investigations, therapies, symptoms, or process of care codes.
3. **No clear mapping class:** All codes included in this class indicate possible IID cases but do not clearly map to IID diagnosis, investigation, or symptom (eg, other viral enteritis).

Codes that refer to chronic conditions or non-intestinal conditions were defined as not mapping to IID and were excluded (eg, *Helicobacter*, *Salmonella* arthritis). We found that case finding was barely affected by the inclusion of codes in the least restrictive “no clear mapping” class and therefore did not use these codes in any analyses.

### Cohort Identification

This study used primary care data recorded during a 52-week period spanning July 2014-July 2015 from the RCGP RSC, a sentinel network representative of the English population [33]. The cohort included patients with a recorded event, registered for the entire period. These data were used to determine the denominator. Data were extracted using SQL (Structured Query Language) software [34].

### Case Finding and Rate Calculations

We calculated case numbers and incidence rates for the three IID definitions. When clinicians record a diagnosis, they assign episode type, which differentiates incident (first, new) cases from prevalent (follow-up, ongoing) cases. Records with “first” or “new” episode types were counted when counting cases and calculating incidence rates using diagnostic codes. When cases were found using directly mapping investigation and process care codes, all episode types were included because it is not standard clinical practice to code these events as “first” or “new.” Patients with excessive IID diagnostic records (>4 per year) were excluded from case counts as they likely had chronic gastrointestinal conditions, although this represented fewer than 10 people over the one-year study period.

### Concepts Supporting Case Finding

We further investigated differences between case definitions and the validity of using an ontological case definition by searching patients who had been already counted as a case for codes relating to four supporting concepts: (1) symptoms (diarrhea, vomiting, and fever), (2) pathology investigations (stool sample sent to laboratory, to test for specific pathogens), (3) process of care (notification of dysentery or food poisoning), and (4) therapies (loperamide or oral rehydration therapy). We used a 2-week sliding window due to IID’s acute nature: all events for supporting concepts recorded with any episode type had to occur 2 weeks before or after the patient’s diagnosis event to be included. In addition, multiple events coded for any one factor within the 2-week window (eg, three investigation codes in one week) were counted as one event. Complete code lists for supporting factors are presented in Multimedia Appendix 2. We counted occurrences of each of the four supporting concepts and created Venn diagrams using R software [34].

The “Integrate” study received a favorable ethical opinion from the NHS NRES Committee North West-Greater Manchester East (Ref: 15/NW/0233). Patient-level data were automatically extracted and pseudonymized at the point of extraction. Data were stored at the University of Surrey Clinical Informatics and Health Outcomes Research Group data and analysis hub such that patients could not be identified from records used during the study.

## Results

We identified an initial cohort (N=1,120,490) used to count cases and calculate incidence rates from the RCGP RSC population among all registered patients with at least one recorded event during a 52-week period spanning ISO 2014-W30 to ISO 2015-W29.

The results of the ontology can be found online (http://webprotege.stanford.edu) under the title “IID infectious intestinal disease ontology.”

**Figure 1:**
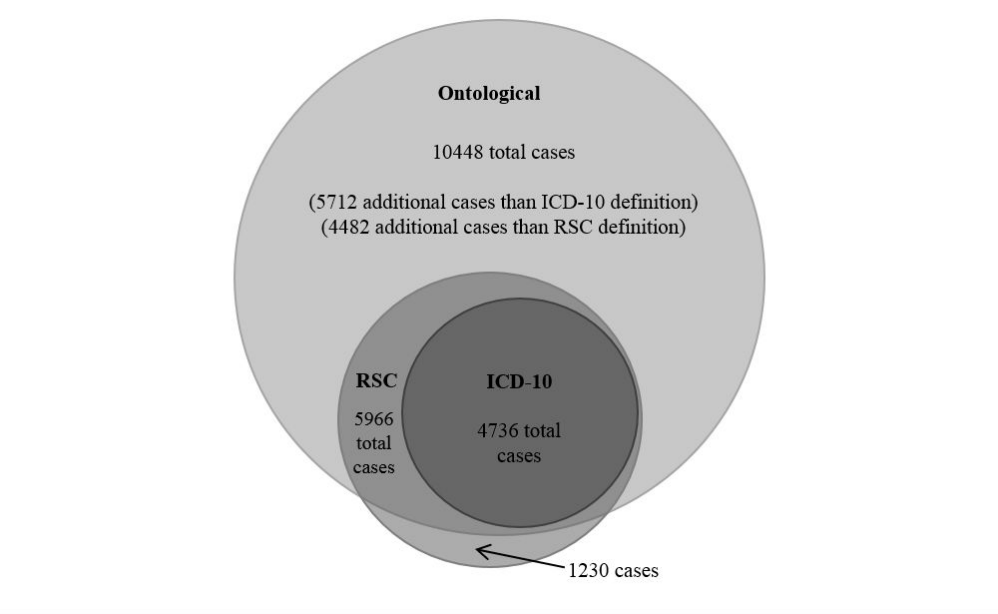
Total number of cases identified using three differing definitions of IID (RSC, ICD-10 and ontological). Cohort includes all registered patients in the RCGP RSC primary care database with at least one recorded event during a 52-week period spanning ISO 2014-W30 to ISO 2015-W29 (initial cohort, N=1120490).

Use of the ICD-10 case definition identified 4736 cases of IID within the cohort, compared with 5966 cases found with the RSC definition (Figure 1).

Application of the ICD-10 definition when selecting Read codes resulted in a more limited code list (90 codes in ICD-9 reduced to 70 codes in ICD-10). This reduction is due to the removal of codes for *Helicobacter* and specific nonintestinal *Salmonella* infections; codes for other specific bacterial and viral infections (Arizona paracolon bacilli, Astrovirus, Calicivirus); and general infection codes that appeared redundant. Until recently, these codes were included in the RSC weekly report which, for consistency in surveillance of disease trends, continued following the ICD-9 system.

A key difference between ICD-10 and RSC weekly report code lists was the inclusion of *Helicobacter pylori* in the RSC definition *,* with 25% (306/1230) of cases captured within the RSC definition being recorded as *Helicobacter* codes. Although this condition is not included in the IID chapter of ICD systems, *H. pylori* infection is included in the IID chapter of the Read code system and therefore has been historically monitored in the RSC weekly report as IID. As *H. pylori* prevalence rates in Europe are at least as high as IID rates [35], its inclusion in IID surveillance could affect disease trend monitoring.

Using the ontological approach, we identified 5712 more cases than the ICD-10 definition and 4482 more cases than the RSC definition within the same cohort (Figure 1). Of the additional ontological cases, 77% (4399/5712) were recorded using specific gastroenteritis codes; 10.2% (582/5712) were coded as diarrhea and vomiting, first or new episodes; and 9.6% (546/5712) were recorded with direct pathology investigation codes (Table 1).

**Table 1:** Counts of additional ontological cases by code type (number of additional ontological cases not included in other case definitions=5712, data for period ISO 2014-W30 to ISO 2015-W29).

Code type	Code	Count of cases	Additional ontological cases (percentage)
Gastroenteritis, toxic gastroenteritis	J43-1 J43..11	4399	77.0
Diarrhea and vomiting	19G%	582	10.2
Clostridium difficile infection	A3Ay2%	145	2.5
Direct pathology investigation	Multiple; see Multimedia Appendix 2	546	9.6
Direct process of care	65V1%, 65V2%	29	0.5

**Table 2:** IID incidence and case counts (Data for period ISO 2014-W30 to ISO 2015-W29, weekly denominator N=1,120,490).

Definition	Count of cases	Annual person-time rates (per 1000 person-time units)
Standard RSC	5966	5.32 (95% CI 5.19-5.46)
ICD-10	4736	4.23 (95% CI 4.11-4.35)
Ontological	10,448	9.32 (95% CI 9.15-9.50)

**Table 3:** Mean weekly incidence rates and case counts (Data for period ISO 2014-W30 to ISO 2015-W29, weekly denominator N=1,120,490).

Definition	Mean weekly count of cases	Incidence rate (per 100,000/week)
Standard RSC	114.73	10.24 (95% CI 8.55-12.12)
ICD-10	91.08	8.13 (95% CI 6.70-9.87)
Ontological	200.92	17.93 (95% CI 15.63-20.41)

Using the ontological definition for case finding resulted in an annual percentage incidence rate of 0.93% (10,448/1,120,490) compared with 0.42% (4736/1,120,490) under the ICD-10 definition and 0.53% (5966/1,120,490) under the RSC definition. Annual person-time rate per 1000 person-time units for the standard RSC definition was 5.32 (95% CI 5.19-5.46), for the ICD-10 definition was 4.23 (95% CI 4.11-4.35), and for the ontological definition was 9.32 (95% CI 9.15-9.50; Table 2).

Mean weekly incidence rate was 10.24 per 100,000 (95% CI 8.55-12.12) for the RSC definition, 8.13 per 100,000 (95% CI 6.70-9.87) for the ICD-10 definition, and 17.93 per 100,000 (95% CI 15.63-20.41) for the ontological definition (Table 3).

**Figure 2:**
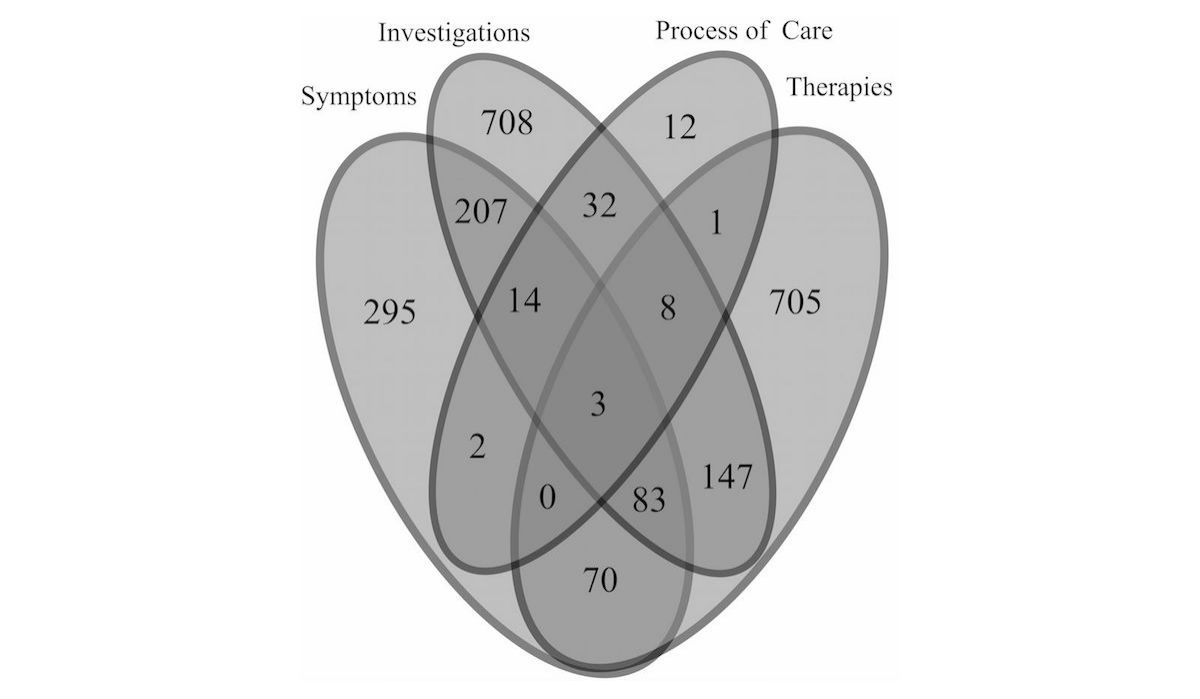
Count of events found using codes for supporting factors (symptom, investigation, process of care, and/or therapy) for cases identified using the standard RCGP RSC IID definition (ISO 2014-W30 to ISO 2015-W29). For Figures 2-4, events found using two-week sliding window: all recorded events for supporting concepts recorded with any episode type had to occur two weeks before or after the patient’s diagnosis event to be included. Multiple events coded for any one factor within the two-week window of the case finding were counted as one event.

**Figure 3:**
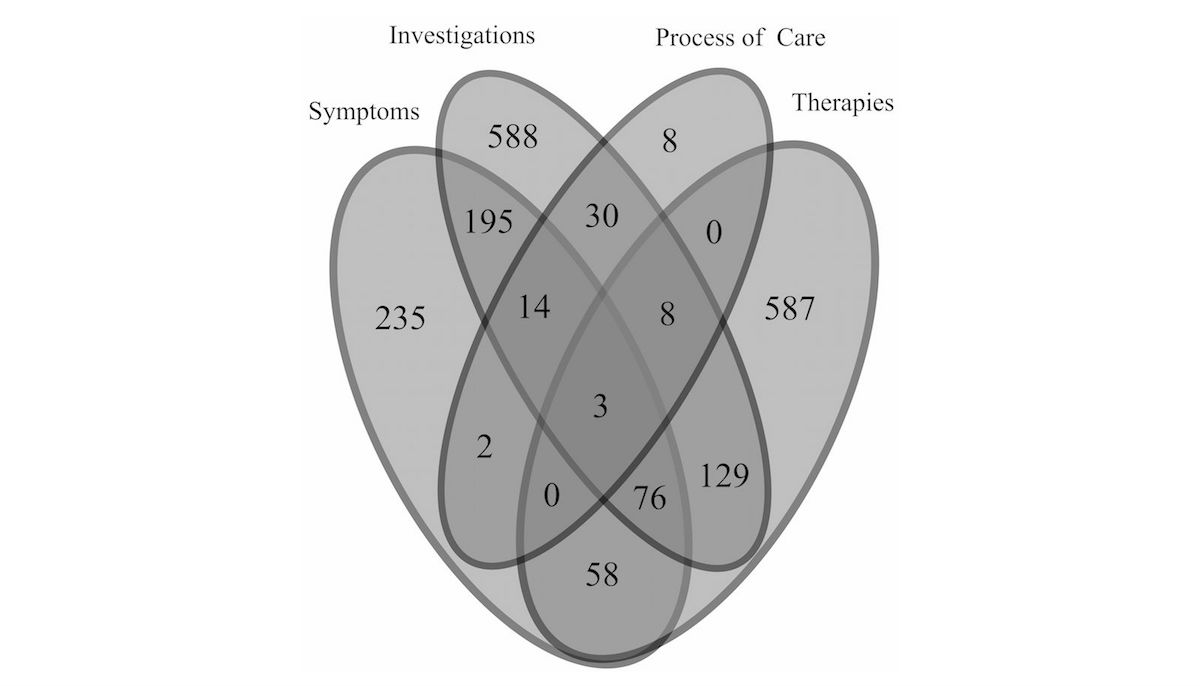
Count of events found using codes for supporting factors (symptom, investigation, process of care, and/or therapy) for cases identified using the ICD-10 IID definition (ISO 2014-W30 to ISO 2015-W29).

**Figure 4:**
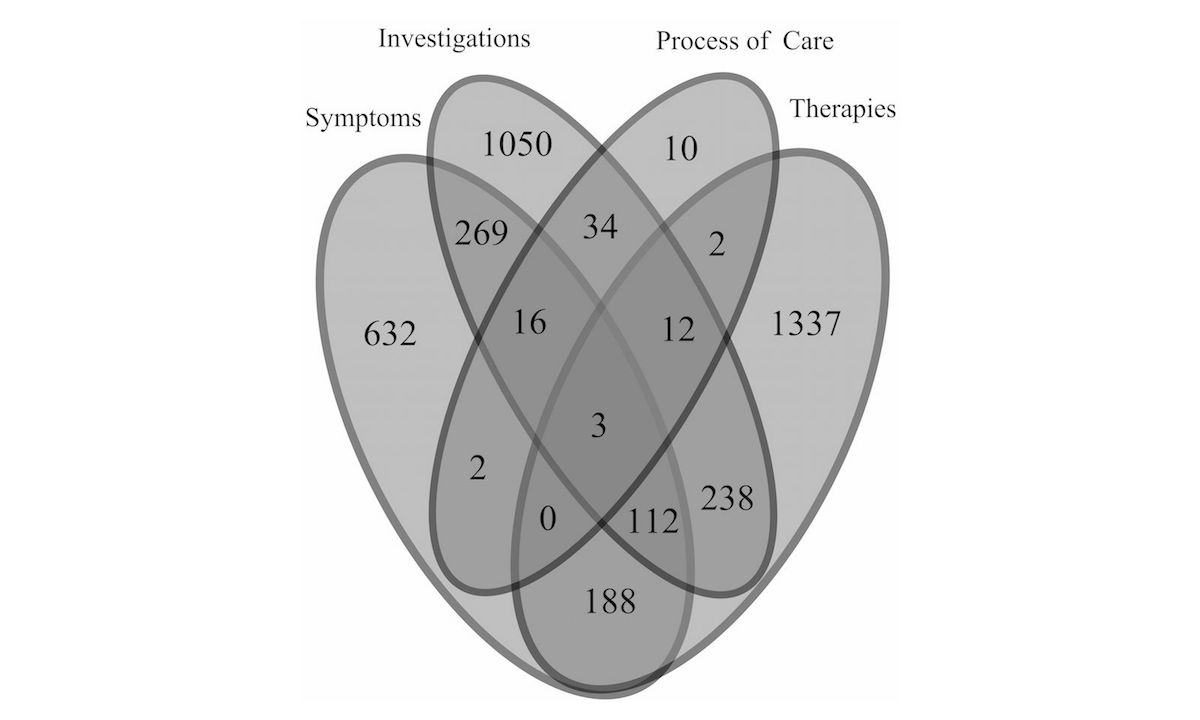
Count of events found using codes for supporting factors (symptom, investigation, process of care, and/or therapy) for cases identified using the ontological IID definition (ISO 2014-W30 to ISO 2015-W29).

Event counts of four supporting concepts within the 2-week period preceding or following case finding were consistent across IID definitions (Figures 2-4,Tables 4-6), with categories differing by ±1-2%.

Consistency of results supports the use of the ontological definition, as supporting concept codes are specific to acute IID. For the three definitions, majority of cases (61.67% [3679/5966], 59.18% [2803/4736], and 62.62% [6543/10,448]) had no supporting concepts recorded within the 2-week sliding window. In addition, proportion of laboratory results associated with positive test results (ie, directly mapping to IID case definition) was 19.7% (546/2775).

**Table 4:** Counts of supporting factors for RSC defined cases (N=5966).

Code category	Number of events coded	Percentage of RSC cases
Symptoms	295	4.94
Investigations	708	11.87
Therapies	705	11.82
Process of care	12	0.20
Symptoms and investigations	207	3.47
Symptoms and therapies	70	1.17
Symptoms and process of care	2	0.03
Investigations and therapies	147	2.46
Investigations and process of care	32	0.54
Therapies and process of care	1	0.02
Symptoms, investigations, and therapies	83	1.39
Symptoms, investigations, and process of care	14	0.23
Symptoms, therapies, and process of care	0	0.00
Investigations, therapies, and process of care	8	0.13
All supporting concepts	3	0.05
Number of cases with any of the above	2287	38.33
Number of cases with none of the above	3679	61.67

**Table 5:** Counts of supporting factors for ICD-10 defined cases (N=4736).

Code category	Number of events coded	Percentage of ICD-10 cases
Symptoms	235	4.96
Investigations	588	12.42
Therapies	587	12.39
Process of care	8	0.17
Symptoms and investigations	195	4.12
Symptoms and therapies	58	1.22
Symptoms and process of care	2	0.04
Investigations and therapies	129	2.72
Investigations and process of care	30	0.63
Therapies and process of care	0	0.00
Symptoms, investigations, and therapies	76	1.60
Symptoms, investigations, and process of care	14	0.30
Symptoms, therapies, and process of care	0	0.00
Investigations, therapies, and process of care	8	0.17
All supporting concepts	3	0.06
Number of cases with any of the above	1933	40.82
Number of cases with none of the above	2803	59.18

**Table 6:** Counts of supporting factors for cases defined ontologically (N=10,448).

Code category	Number of events coded	Percentage of ontological cases
Symptoms	632	6.05
Investigations	1050	10.05
Therapies	1337	12.80
Process of care	10	0.10
Symptoms and investigations	269	2.57
Symptoms and therapies	188	1.80
Symptoms and process of care	2	0.02
Investigations and therapies	238	2.28
Investigations and process of care	34	0.33
Therapies and process of care	2	0.02
Symptoms, investigations, and therapies	112	1.07
Symptoms, investigations, and process of care	16	0.15
Symptoms, therapies, and process of care	0	0.00
Investigations, therapies, and process of care	12	0.11
All supporting concepts	3	0.03
Number of cases with any of the above	3905	37.38
Number of cases with none of the above	6543	62.62

## Discussion

### Principal Findings

An ontological approach to IID case finding changed IID incidence rate, increasing case detection. The ontological approach is also more transparent and independent of coding systems.

The ontological approach may address elements of IID underestimation due to low rates of case finding using electronic data alone [36], depending upon the case definition used [8,15,37]. However, the major limitation to accurate case finding remains that many community cases of IID do not seek health care [38].

GPs appear more likely to enter symptom codes, which from the ontological perspective are less helpful as they overlap with other conditions rather than being specific to IID, unless the symptoms are supported by another code indicating pathogen detection [39]. Results of the ontological approach have highlighted how use of symptom codes contributes to underreporting IID patients who do not have appropriate diagnostic or surveillance codes entered into the patient record.

### Implications of Findings for Clinical Practice

An ontological approach provides insights into what types of data are available for case ascertainment. Although this approach offers benefits, and has limitations, our recommendation is to start by making the laboratory results recorded much more specific.

The mechanism for transferring results from stool sampling to GPs needs to be updated. Currently UK laboratories electronically report stool sample results to GPs using the Pathology Bounded Code List (PBCL), a subset of Read codes. However, there is no standardized algorithm for reporting results, and the PBCL code list for Microscopy, Culture & Sensitivity (MC&S) results has not kept pace with developments in pathology services. For example, typical laboratory protocol is to report one generic stool sampling code per test request, regardless of the range of pathogens being screened or detected, or of the sensitivity or specificity of the testing method. When a GP receives electronic results of a stool sample, the electronic report only contains generic MC&S Read codes, indicating that a stool sample was analyzed. This is followed by a “free-text” message (ie, not coded) indicating any detected pathogens. If pathogens are detected, the clinician must then code this information manually into the computerized medical record (CMR) system. This means that, inevitably, laboratory findings are under-coded. Furthermore, for some pathogens there is only one PBCL code specifically for test requests, not for recording results. Many IID pathogens have no designated PBCL code at all, and where appropriate pathogen codes are available, they are often not used. Given likely advances in stool microbiology testing in the future, with a move away from MC&S to nonculture, PCR-based methods, the way microbiology results are reported and coded via PBCL needs to be reviewed and modernized. There might be scope to draw lessons from biochemistry and hematology where, with the exception of glucose provenance and use of nonnumeric keys [39] and the use of nonnumeric characters, results with coded data are generally readily filed into the CMR system.

### Limitations

The principal limitation of this study is the lack of a gold standard; we do not know the “true” incidence of IID. There has been no back-to-case records review to validate this approach, though the authors have gone back to records to demonstrate the reliability of case finding from clinical records in other domains, for example, chronic kidney disease [40] and diabetes [41,42]. We have also reported where we consider conclusions to be unsafe because the wrong codes were selected [43].

In addition, ontologies are developed as an iterative process; therefore, we recommend testing by running data extracts to improve sensitivity and specificity. Our ontology is online and may be superseded by better laboratory coding, advances in near-patient testing, or other unforeseen advances. For example, there was no attempt to include social media data in this exercise. Techniques are emerging to do this and should be considered as part of future investigations and for inclusion in the subjective elements of the ontology [44,45].

Bias of many types can affect the quality of data recording in CMR systems. This can be around financial incentives to adopt CMR systems which then may not get used [46]; and around pressures within systems to either investigate, refer, or prescribe more (or less) depending on the constraints within the individual health care system at the time. These effects are probably best reported for drug safety studies where the availability of a large number of CMR records or administrative datasets had not obviated the need for other mechanisms of drug safety recording [47,48].

Finally, use of a new ontological approach to measuring disease incidence might result in further discrepancies between different surveillance systems that monitor the IID incidence. Harmonization of coding systems across different systems and countries is important from an epidemiological perspective to ensure that estimates of disease burden are comparable.

### Conclusions

Our study indicates that use of the standard definition of IID to identify cases in primary care results in the underestimation of disease incidence. To capture a larger proportion of new IID cases in primary care, an ontological approach should be adopted to expand the case definition to include those patients with codes falling outside more restrictive standard definitions, as well as improving the PBCL coding list used by laboratories returning pathology results. Given the high burden of IID in the community, identifying what specific organisms are circulating within a community would help GPs and public health services. For GPs this would reinforce the importance of stressing simple and important control measures, such as hand washing, and trigger the implementation-specific interventions for specific infections. Local and regional public health services would more accurately know the disease burden and be able to intervene; nationally and internationally more accurate data would enable better policy evaluation and development around hygiene and food chain management.

Using these approaches will provide a better picture for clinicians, epidemiologists, and public health officials of the burden of IID in the community and the impact of seasonal infectious disease outbreaks.

## Appendix

Multimedia Appendix 1 Formal ontology developed based on infectious intestinal disease case definition.

Multimedia Appendix 2 Diagnostic and supporting factor codes and code mapping classes selected based on infectious intestinal disease ontology.

## Abbreviations

CMR: computerized medical record
GP: general practitioner
ICD: International Classification of Diseases
IID: infectious intestinal disease
MC&S: Microscopy, Culture & Sensitivity
PBCL: Pathology Bounded Code List
RCGP: Royal College of General Practitioners
RSC: Research and Surveillance Centre
WHO: World Health Organisation
